# Genome sequences of 15 *Escherichia coli* O77g strains related to an emerging STEC lineage

**DOI:** 10.1128/mra.00194-26

**Published:** 2026-04-27

**Authors:** Mai-Lan Tran, Fabien Vorimore, Patrick Fach, Sabine Delannoy

**Affiliations:** 1 Pathogenic E. coli Unit (COLiPATH), Laboratory for Food Safety, Anses, Maisons-Alfort, France; 2 IdentyPath Genomics Platform (IDPA), Laboratory for Food Safety, Anses, Maisons-Alfort, France; University of Maryland Baltimore, Baltimore, Maryland, USA

**Keywords:** Shiga toxins, *Escherichia coli*

## Abstract

We report the genome sequences of 15 *Escherichia coli* O77g strains belonging to the O17, O44, O73, O77, and O106 antigen cluster. Strains of *E. coli* O77g have been associated with hemolytic uremic syndrome (HUS) cases in France in early 2025.

## ANNOUNCEMENT

Shiga toxin-producing *Escherichia coli* (STEC) are major foodborne pathogens responsible for sporadic and epidemic cases of hemolytic uremic syndrome (HUS) worldwide (1). In January 2025, HUS cases were reported in France and linked to unpasteurized cheese contaminated with an emerging STEC O77g:H18 belonging to sequence type 69 (ST69) (*stx2d*-positive, *eae*-negative) (2). To provide comparative genomics and molecular epidemiology of this lineage, 15 *E. coli* O77g isolates from our lab collection were sequenced and assembled. These strains belong to serogroups O17, O44, O73, O77, and O106, which share the same O-antigen gene cluster (3). *E. coli* strains were originally isolated on trypticase soy broth with yeast extract (TSYE) plates overnight at 37°C. Prior to DNA extraction, a colony was grown overnight in brain heart infusion (BHI) broth at 37°C with agitation. Genomic DNA was extracted from 1 mL of culture using the DNeasy Blood and Tissue Kit (Qiagen, France) according to the manufacturer’s instructions. All 15 isolates were sequenced on R10.4.1 flow cells using a MinION Mk1D device (Oxford Nanopore Technologies, Oxford, UK). Libraries were prepared with 200 ng extracted DNA using the Rapid Barcoding Kit SQK-RBK114.24 (Oxford Nanopore Technologies) following the manufacturer’s protocol. DNA was not sheared nor size selected prior to library preparation. Sequencing runs were performed for 72 h using MinKNOW v25.09.16, and raw data were live basecalled using the Super-accurate model v5.2.0-400bps. The option “trim barcodes,” “barcode both ends,” and “Min Q score 10” was enabled on the MinKNOW (4). Raw reads from the pass folder were trimmed using chopper v0.10.1 (5) with “-l 1000” parameter to filter reads below 1,000 bp length. The min quality parameter was set to 20 and 25 for the *E. coli* O17:H18 reference strain K12a and the LP-26-E1-261 strain, respectively. Assemblies of the strains were generated using autocycler v0.5.0 (6) with the custom script provided by the author (https://github.com/rrwick/Autocycler/blob/main/pipelines/Automated_Autocycler_Bash_script_by_Ryan_Wick/autocycler_full.sh).

Assemblies were corrected using Medaka v2.0.1 (https://github.com/nanoporetech/medaka) using the “–bacteria” option (model r1041_e82_400bps_bacterial_methylation). Statistics on raw reads were obtained using NanoPlot v1.45.0 (4). A total of 37,760 to 447,721 reads were generated with *N*_50_ values ranging from 6,125 to 14,398 bp (mean, 12,130.40 ± 2,189.38 bp). The number of contigs per assembly ranged from one to nine with total assembly lengths between 4,879,178 and 5,580,222 bp. The mean GC content was 50.58% (±0.12). Assembly *N*_50_ values ranged from 4,879,178 to 5,244,335 bp. All genomes submitted were circular, chromosome, and plasmids, if any, but they were not rotated to a specific base. The number of contigs was defined using the output of autocycler “consensus_assembly.yaml” that counts the number of contigs and indicates the topology. All contigs were complete and circular, so the number of contigs in Table 1 indicates one chromosome and the rest are plasmids, if any. Detailed strain information, including serotype, presence of *stx* and *eae* genes, genome coverage, and accession numbers is reported in Table 1. All genomes were annotated using the National Center for Biotechnology Information (NCBI) Prokaryotic Genome Annotation Pipeline (PGAP) v6.10 (7) at https://www.ncbi.nlm.nih.gov/refseq/annotation_prok/.

**TABLE 1:** Assembly metrics and accession numbers for the 15 *Escherichia coli* O77g isolates sequenced.

Strain	Source niche	Source type	Source details	Collection year	Continent	Country	ST^*a*^	ST complex^*a*^	Serotype^*b*^	eae^*c*^	stx^*d*^	Phylogroup^*e*^	Number of raw reads	MinION read length (*N*_50_ [bp])	No. of contigs	Genome size (bp)	Assembly *N*_50_ (bp)	GC (%)	Genome coverage	NCBI accession no. of assembly	NCBI accession no. of reads
LP-26-E1-261	Environment	Water	River	2018	Europe	France	69	ST69 Cplx	O77g:H18	–^*g*^	–	D	161,560	6,125	9	5,580,222	5,039,650	50, 76	130	JBVDMR000000000	SRR37276457
ECA64	Livestock	Bovine	Dairy product	1995	Europe	France	663	–	O77g:H18	–	stx2d	D	264,842	9,098	3	5,233,331	5,059,963	50, 71	296	JBVDMQ000000000	SRR37276456
CB209	Human	Human	*Homo sapiens*; stool	1987	Europe	Germany	449^*f*^	–	O77g:H18	–	–	D	116,374	12,099	3	5,222,438	5,089,256	50, 53	170	JBVDMP000000000	SRR37276450
CB6837	Human	Human	*Homo sapiens*	1997	Europe	Germany	663	–	O77g:H18	–	stx1a, stx2a	D	235,219	11,945	3	5,314,391	5,135,902	50,7	329	JBVDMO000000000	SRR37276449
CB6886	Human	Human	*Homo sapiens*	1997	Europe	Germany	69	ST69 Cplx	O77g:H18	–	–	D	113,334	12,507	5	5,250,473	5,040,535	50, 76	160	JBVDMN000000000	SRR37276448
CB7286	Human	Human	*Homo sapiens*	1997	Europe	Germany	69	ST69 Cplx	O77g:H18	–	–	D	142,169	11,726	5	5,484,097	5,244,335	50, 67	174	JBVDMM000000000	SRR37276447
CB7453	Human	Human	*Homo sapiens*	1998	Europe	Germany	69	ST69 Cplx	O77g:H18	–	–	D	104,279	14,351	4	5,131,675	4,923,747	50, 53	176	JBVDML000000000	SRR37276446
CB8671	Human	Human	*Homo sapiens*	2001	Europe	Germany	69	ST69 Cplx	O77g:H18	–	–	D	145,483	11,161	2	5,046,815	4,921,061	50, 62	195	JBVDMK000000000	SRR37276445
DG341-1	Poultry	Poultry	Chicken	1990	Europe	Germany	69	ST69 Cplx	O77g:H18	–	–	D	139,310	13,069	4	5,410,318	5,067,659	50, 45	207	JBVDMJ000000000	SRR37276444
DG342-2	Poultry	Poultry	Chicken	1990	Europe	Germany	69	ST69 Cplx	O77g:H18	–	–	D	37,760	13,518	4	5,410,318	5,067,659	50, 45	56	JBVDMI000000000	SRR37276443
DG343-1	Poultry	Poultry	Chicken	1990	Europe	Germany	69	ST69 Cplx	O77g:H18	–	–	D	126,418	14,176	4	5,410,318	5,067,659	50,45	198	JBVDMH000000000	SRR37276455
DG351-2	Poultry	Poultry	Chicken	1990	Europe	Germany	69	ST69 Cplx	O77g:H18	–	–	D	160,270	11,356	4	5,410,318	5,067,659	50, 45	204	JBVDMG000000000	SRR37276454
KK05-19	Human	Human	*Homo sapiens*; stool	1986	Europe	Germany	130	ST31 Cplx	O77g:H18	–	–	D	104,452	13,741	1	4,879,178	4,879,178	50, 61	176	JBVDMF000000000	SRR37276453
KK19-02	Human	Human	*Homo sapiens*; stool	1986	Europe	Germany	130^*f*^	–	O77g:H4	–	–	G	144,217	12,686	2	5,324,074	5,137,557	50, 57	209	JBVDME000000000	SRR37276452
O17_K12a	Human	Human	*Homo sapiens*; stool	1940	Europe	Denmark	925	–	O17:H18	–	–	D	447,721	14,398	3	5,272,206	5,093,858	50, 47	751	JBVDMD000000000	SRR37276451

^*a*^ Determined using MLST v2.0 (with the *E. coli* #1 MLST scheme and database version 2023-06-19) on the CGE website (https://www.genomicepidemiology.org/).

^*b*^ Determined using Abricate (https://github.com/tseemann/abricate) with the Ecoh database (8).

^*c*^ Determined using Abricate (https://github.com/tseemann/abricate) with VirulenceFinder.

^*d*^ Determined using Abricate and a custom database (https://bitbucket.org/genomicepidemiology/virulencefinder_db/src/master/stx.fsa).

^*e*^ Determined using EzClermont v0.7.0 (https://github.com/nickp60/EzClermont).

^*f*^ Presence of a novel allele. Nearest ST is displayed.

^*g*^ –, not available.

## Data Availability

The genome assemblies and corresponding raw reads have been deposited in the NCBI database under BioProject accession number PRJNA1424694.
